# Airborne Bacterial and Eukaryotic Communities in Temuco, Chile, a City Dominated by Residential Wood Combustion

**DOI:** 10.3390/life16091433

**Published:** 2026-08-28

**Authors:** Juan Liu, So Fujiyoshi, Fumito Maruyama, Jun Noda, Tay Ruiz-Gil, Elizabeth Carrazana, Jacquelinne J. Acuña, Milko A. Jorquera, Nobutake Nakatani, Shiyuan Guo, Akihiro Sakatoku, Daisuke Tanaka

**Affiliations:** 1 Graduate School of Science and Engineering, University of Toyama, Toyama 930-8555, Japan; d24c8305@ems.u-toyama.ac.jp (J.L.); d25c8303@ems.u-toyama.ac.jp (S.G.); sakatoku@sci.u-toyama.ac.jp (A.S.); 2 Microbial Genomics and Ecology, the IDEC Institute, Hiroshima University, Hiroshima 739-8530, Japan; sofu62@hiroshima-u.ac.jp (S.F.); fumito@hiroshima-u.ac.jp (F.M.); 3 Department of Environmental and Civil Engineering, Faculty of Engineering, Toyama Prefectural University, Toyama 939-0398, Japan; 4 Graduate School of Veterinary Medicine, Rakuno Gakuen University, Ebetsu 069-8501, Japan; jnoda@rakuno.ac.jp; 5 Laboratorio de Ecología Microbiana Aplicada, Departamento de Ciencias Químicas y Recursos Naturales, Universidad de La Frontera, Temuco 4811230, Chile; tayruizbio@gmail.com (T.R.-G.); eli.carrazana97@gmail.com (E.C.); jacquelinne.acuna@ufrontera.cl (J.J.A.); milko.jorquera@ufrontera.cl (M.A.J.); 6 Centro de Investigación, Innovación y Creación, Vicerrectoría de Investigación y Posgrado, Universidad Católica de Temuco, Temuco 4813302, Chile; 7 Laboratory of Neurodegenerative Diseases, Center for Biomedicine, Universidad Mayor, Temuco 4801043, Chile; 8 Department of Environmental Sciences, College of Agriculture, Food and Environment Sciences, Rakuno Gakuen University, Ebetsu 069-8501, Japan; nakatani@rakuno.ac.jp

**Keywords:** residential wood combustion, particulate matter, bioaerosols, microbial communities, water-soluble inorganic ions (WSIIs)

## Abstract

Airborne particulate matter (PM) affects human health, especially in heavily polluted regions. This study focused on Temuco, a city dominated by residential wood combustion in southern Chile. For the first time, we characterized airborne eukaryotic microbial communities in this area, alongside a parallel analysis of bacterial communities, and explored their associations with water-soluble inorganic ions (WSIIs) and meteorological variables. Bacterial and eukaryotic communities were profiled using 16S and 18S rRNA gene sequencing, respectively. The results showed that the mean concentrations of PM_2.5_ and PM_10_ were 75.0 and 87.7 μg/m^3^, respectively, with PM_2.5_ exceeding the Chilean national air quality standards (PM_2.5_: 50 μg/m^3^; PM_10_: 130 μg/m^3^), highlighting the persistent fine particulate matter pollution in the region. Among the WSIIs, NO_3_^−^ (5.6 μg/m^3^), SO_4_^2−^ (2.6 μg/m^3^), and K^+^ (2.6 μg/m^3^) were the most abundant, indicating important contributions from secondary aerosol formation and residential biomass burning. At the genus level, *Clostridium* (6.2%), *Hymenobacter* (4.3%), and *Sphingomonas* (4.3%) dominated the bacterial communities, whereas *Coriolopsis* (41.0%) and *Hyphodontia* (26.9%) were the predominant eukaryotic taxa. Community composition differed between seasons and particle-size fractions, with seasonal variation appearing to be more pronounced. Correlation analysis and redundancy analysis (RDA) further showed relatively high loadings of SO_4_^2−^ (loading = 0.7146) and temperature (Temp, loading = 0.7101) in the bacterial RDA, and of Cl^−^ (loading = −0.9054) and SO_4_^2−^ (loading = −0.7176) in the eukaryotic RDA. Overall, these findings provide important insights into the ecology of atmospheric microorganisms and their potential environmental associations in a city dominated by residential wood combustion.

## 1. Introduction

Bioaerosols are widely distributed in the atmosphere and are primarily composed of microorganisms (such as bacteria, fungi, viruses, and algae), spores of mosses and ferns, pollen, plant debris, and animal residues [1]. The transport and dispersion of airborne microorganisms play a crucial role in ecological processes, public health, and atmospheric dynamics, as they not only facilitate microbial exchange across different environments but also contribute to the regional spread of pathogens. In addition, these microorganisms participate in cloud formation and precipitation processes [2,3] and promote microbial colonization in pristine or remote environments [4]. It is well established that genera such as *Neisseria*, *Corynebacterium*, and *Bacillus* contain important pathogenic species responsible for diseases including meningitis, diphtheria, and anthrax, respectively [5,6,7]. Compared with non-pathogenic bacteria, pathogenic microorganisms often exhibit greater tolerance to airborne environmental stressors and toxic substances [8].

In recent years, airborne eukaryotic microbial communities have attracted growing research interest due to their significant roles in atmospheric ecosystems and public health. These microorganisms can serve as important aeroallergens, with certain taxa linked to respiratory diseases and other adverse health outcomes. In particular, eukaryotic genera such as *Alternaria*, *Aspergillus*, *Cladosporium*, and *Penicillium* have been reported to be associated with asthma exacerbation, asthma hospitalization, and hypersensitivity pneumonitis [9]. In addition, some airborne fungal spores can survive for months under suitable conditions, thereby facilitating their persistence and transport in the atmosphere and potentially increasing human exposure risks [10].

Airborne particulate matter (PM) can act as a carrier of pathogenic microorganisms, facilitating their survival and deposition in the human respiratory tract. Particles of different sizes play distinct roles in microbial enrichment and transport. Fine particles (PM_2.5_) can penetrate deeply into the lungs, whereas coarse particles (PM_10_) are more likely to harbor microbial communities originating from soil and vegetation [11]. The interactions between chemical and biological components in particulate matter may enhance the adverse effects of PM on the human respiratory system by inducing inflammatory responses and oxidative stress in the respiratory tract [12]. Moreover, the synergistic effects of chemical and biological constituents can further exacerbate health risks, potentially contributing to chronic respiratory diseases, asthma, and lung cancer [13]. For instance, water-soluble inorganic ions (WSIIs) can influence microbial survival, attachment, and metabolism by altering particle pH, nutrient availability, and moisture conditions, thereby shaping microbial community composition [14]. Seasonal variations and associated meteorological conditions shape airborne microbial communities. Factors such as temperature, relative humidity, and air pollution levels can significantly influence the composition and concentration of bioaerosols [15,16].

In recent years, the development of culture-independent microbial analysis techniques, particularly DNA-based molecular approaches, has greatly advanced the comprehensive characterization of atmospheric microbial communities [17]. Building on these advances, extensive studies have been conducted worldwide to investigate the diversity and community composition of airborne microorganisms associated with PM [18,19]. Temuco, located in southern Chile, is a representative biomass-burning city that suffers from severe PM pollution. Together with the neighboring city of Padre Las Casas, Temuco has been officially designated as a saturated zone for both PM_10_ and PM_2.5_. During the winter heating season, intensive residential wood combustion, combined with low temperatures, atmospheric inversions, and poor dispersion conditions, promotes the accumulation of PM, with PM_2.5_ accounting for up to 89.9% of PM_10_ during certain periods. According to official emission inventories, residential wood combustion accounts for approximately 91.2% of PM_2.5_ emissions, establishing it as the primary source of air pollution in the region [20,21]. Although previous studies have provided some insights into particulate matter-associated bacterial communities in this region [22,23], research specifically targeting airborne eukaryotic microorganisms has not yet been conducted in this region. Moreover, relatively few studies have examined the influences of seasonal variation, particle-size fractions, and aerosol chemical composition on airborne bacterial and eukaryotic communities in a city dominated by residential wood combustion.

Therefore, in this study, molecular biological approaches were employed to systematically investigate the composition, structure and diversity of bacterial and eukaryotic communities in Temuco, Chile. The specific objectives were as follows: (1) characterize the concentrations and seasonal variations in PM_2.5_, PM_10_ and WSIIs; (2) investigate the composition and diversity of airborne bacterial and eukaryotic communities associated with PM during winter and spring; (3) evaluate the relationships between microbial communities and environmental factors and explore potential environmental factors associated with community structures. This study provides insights into the characteristics, seasonal dynamics, and potential environmental associations of airborne bacterial and eukaryotic communities in a city dominated by residential wood combustion.

## 2. Materials and Methods

### 2.1. Air Samples

PM_2.5_ and PM_10_ samples were collected in Temuco, Chile, between June and October 2021 using the air quality monitoring (AQM) system equipped with a low-volume air sampler and a beta-attenuation monitor (BAM, Model 1020, Met One Instruments, Inc., Grants Pass, OR, USA) with glass-fiber filter tape. Sampling was conducted at a suburban site on the roof of a three-story building at the Universidad de La Frontera (38°44′45.2″ S, 72°36′58.4″ W, 130 m above mean sea level, AMSL). Under the standardized AQM protocol, air was pumped at a flow rate of 16.7 L/min through the filter tape, and each PM fraction was collected continuously for 1 h; 24 consecutive sampling spots were used to represent one sampling day. After sampling, the filter tape was transported to the laboratory and processed for DNA extraction. Temuco is the capital of Cautín Province and the Araucanía Region in southern Chile. The city has a mild oceanic climate with distinct seasonal variations. Winters from June to August are generally mild and wet, whereas spring, from September to November, is characterized by gradually increasing temperatures and longer days.

Meteorological parameters, including temperature (Temp), relative humidity (RH), and wind speed, as well as PM_2.5_ and PM_10_ concentrations, were obtained from the local air quality monitoring station operated by the Chilean National Air Quality Information System (SINCA, https://sinca.mma.gob.cl). The data corresponded to the sampling days and represented daily averages. A summary of the data is provided in Table 1. Although these data were obtained from the nearest monitoring station rather than directly at the sampling site, they were considered representative of the general meteorological conditions during the sampling period.

### 2.2. 16S and 18S rRNA Gene Amplicon Sequencing

The filter samples were processed using the ISOSPIN Soil DNA extraction kit (Nippon Gene Co., Ltd., Tokyo, Japan) following the manufacturer’s instructions. To prevent potential contamination, DNA extraction and PCR preparation were carried out in a laminar airflow clean bench. For bacterial community analysis, the V3–V4 region of the 16S rRNA gene was amplified because this region offers broad bacterial coverage, high taxonomic resolution, and reliable representation of microbial diversity [24]. PCR amplification was conducted with primers 341F and 805R according to the Illumina “16S Metagenomic Sequencing Library Preparation” protocol (Illumina Inc., San Diego, CA, USA, Part #15044223 Rev. B). The cycling conditions consisted of an initial denaturation at 94 °C for 2 min; 25–35 cycles of denaturation at 98 °C for 10 s, annealing at 55 °C for 30 s, and extension at 68 °C for 30 s; and a final extension at 68 °C for 7 min. For eukaryotic community analysis, the V8 region of the 18S rRNA gene was amplified using the primer pair 1422f and 1642r. The V8 region was selected because it minimizes the co-amplification of non-target DNA and provides improved specificity and taxonomic resolution for diverse eukaryotic taxa in environmental samples [25]. The PCR cycling conditions consisted of an initial denaturation at 94 °C for 2 min, followed by 35 cycles of denaturation at 94 °C for 30 s, annealing at 55 °C for 30 s, and extension at 72 °C for 30 s; with a final extension at 72 °C for 5 min. A negative control, consisting of all PCR reagents except template DNA, was included in the PCR reactions. PCR reactions and Illumina MiSeq (Illumina, San Diego, CA, USA) sequencing were performed as described previously [26,27]. The PCR products were purified using Agencourt AMPure XP beads (Beckman Coulter, CA, USA). DNA quantifications were performed using a Synergy H1 (BioTek, Tokyo, Japan) and the QuantiFluor dsDNA System (Promega, Madison, WI, USA).

Purified amplicons were pooled in equimolar amounts and subjected to paired-end sequencing (2 × 300 bp) on an Illumina MiSeq instrument (Illumina) with the MiSeq reagent kit V3 (Illumina) according to standard protocols. Quality filtering was performed using the Fastx Toolkit version 0.0.14. Using the DADA2 plugin in QIIME 2 (version 2022.2), primer sequences were removed, and 120 bp were trimmed from the 3′ ends of the reads. Chimeric and noise sequences were removed, and representative sequences and the ASV table were generated. Taxonomic assignment was performed using Greengenes version 13_8 for the 16S rRNA gene and SILVA version 132 for the 18S rRNA gene. For 16S rRNA gene amplicon analysis, singleton ASVs and ASVs assigned to chloroplasts and mitochondria were removed. Statistical analyses were conducted using R (version 4.4.2). All sequences were deposited in the DNA Data Bank of Japan (DDBJ) under the accession number PRJDB42522. Field blanks were subjected to the same extraction and sequencing procedures as the environmental samples to monitor contamination associated with field handling (Tables S1 and S2). No formal contaminant-filtering procedure was applied.

### 2.3. Determination of Water-Soluble Inorganic Ions

The filters were cut into 1 cm^2^ pieces, and six filter pieces were combined and placed in 12-mL polypropylene tubes (Maruemu Co., Osaka, Japan) to avoid contamination by glass-derived elements (e.g., Si and Al). The samples were extracted with 4 mL of deionized distilled water (Auto Still WG262, Yamato Scientific, Tokyo, Japan) and sonicated for 20 min at room temperature. The extracts were filtered through 0.45 µm syringe filters (Sartorius, Göttingen, Germany) to remove coarse particles.

Ion chromatography (IC) was used to quantify inorganic ions. A total of 13 ionic species were analyzed. Anions (F^−^, Cl^−^, NO_2_^−^, Br^−^, NO_3_^−^, PO_4_^3−^, and SO_4_^2−^) were measured using an IC-20 system (Dionex, Sunnyvale, CA, USA) with an IonPac AS12A column and AERS 500 suppressor. The eluent consisted of 2.7 mM Na_2_CO_3_ and 0.3 mM NaHCO_3_, with a flow rate of 1.5 mL/min, a column temperature of 30 °C, and an injection volume of 25 µL. Cations (Li^+^, Na^+^, NH_4_^+^, K^+^, Mg^2+^, and Ca^2+^) were analyzed using an IC-2010 system (Tosoh, Tokyo, Japan) with a TSKgel Super IC-CR column. The eluent (2.2 mM methanesulfonic acid with 1.0 mM 18-crown-6 ether) was delivered at 0.8 mL/min, with a column temperature of 45 °C and an injection volume of 30 µL. Calibration was performed using commercial multi-ion standards (FUJIFILM Wako, Osaka, Japan). Calibration curves showed good linearity (R^2^ > 0.995 for anions and >0.997 for cations). Method detection limits ranged from 0.012 to 0.12 mg/L for anions and 0.007 to 0.027 mg/L for cations, with analytical uncertainties between 2% and 8%.

### 2.4. Statistical Analysis

Statistical analyses were performed to examine the relationships between microbial communities and environmental variables. Correlation analyses were conducted in R (version 4.4.2), and Spearman’s rank correlation coefficients were calculated to assess associations between microbial taxa and environmental variables. Given the limited sample size (*n* = 11), *p* values were adjusted using the Benjamini–Hochberg (BH) procedure to control the false discovery rate (FDR) and reduce the likelihood of false-positive results [28]. Detailed BH-adjusted *p* values are provided in Tables S3–S6.

Prior to redundancy analysis (RDA), detrended correspondence analysis (DCA) was performed to evaluate the gradient length of the species data. The first-axis gradient lengths were 1.41 for bacterial communities and 1.97 for fungal communities, both below 3 standard deviations (SD), indicating that a linear ordination method (RDA) was appropriate [29]. Variance inflation factor (VIF) analysis was performed to reduce multicollinearity among environmental variables. Variables with VIF values >10 were sequentially removed until all remaining variables had VIF values ≤ 10 [30]. RDA was then conducted to evaluate the relationships between environmental variables and microbial community structure. The significance of the overall RDA models, canonical axes, and environmental variables was assessed using permutation tests with 999 permutations. Detailed RDA results are presented in Tables S7–S10. Statistical significance was defined as *p* < 0.05.

## 3. Results and Discussion

### 3.1. Characteristics of Particulate Matter and Water-Soluble Inorganic Ions

The mean concentrations of PM_2.5_ and PM_10_ in Temuco were 75.0 and 87.7 μg/m^3^, respectively. Compared with the Chilean national air quality standards, the average PM_2.5_ concentration exceeded the daily standard of 50 μg/m^3^, whereas the average PM_10_ concentration remained below the corresponding limit of 130 μg/m^3^ [21]. Clear seasonal variations were observed for both particulate fractions. During winter, the mean concentrations of PM_2.5_ and PM_10_ reached 104.6 and 118.1 μg/m^3^, respectively, while substantially lower concentrations were recorded in spring (Welch’s two-sample *t*-test; PM_2.5_, *p* = 0.006; PM_10_, *p* = 0.010), with mean values of 23.3 μg/m^3^ for PM_2.5_ and 34.5 μg/m^3^ for PM_10_. These results indicate that particulate matter pollution in Temuco was markedly more severe in winter than in spring. This pronounced seasonal pattern was likely driven by intensive residential wood combustion during the heating season, together with low temperatures, thermal inversions, and poor atmospheric dispersion conditions that favor the accumulation of particulate matter. The slightly lower PM_10_ concentration than PM_2.5_ observed in Sample 11 (Table 1) may reflect measurement uncertainty in the BAM monitoring system rather than a true difference between the two particulate matter fractions. Because the difference was small (approximately 3 μg/m^3^), the original monitoring data were retained for subsequent analyses.

**Figure 1:**
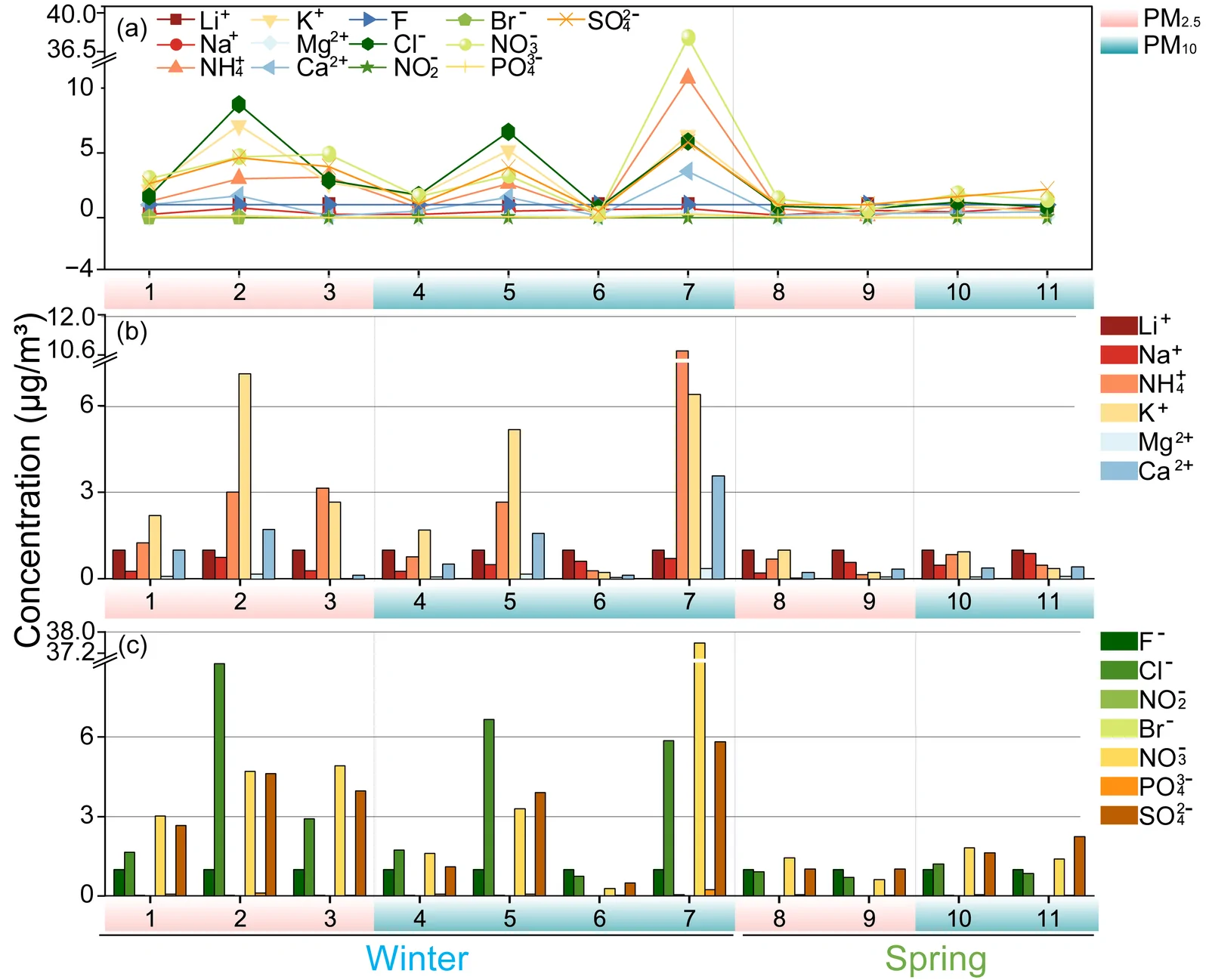
Seasonal variations in WSIIs concentrations in PM_2.5_ and PM_10_. (**a**) total concentrations of all detected WSIIs; (**b**) concentrations of major cations; and (**c**) concentrations of major anions during the winter and spring sampling periods. The numbers on the x-axis represent the sampling order corresponding to the dates listed in Table 1.

The mass concentration distribution of 13 WSIIs is shown in Figure 1. Among cations, K^+^ and NH_4_^+^ were the dominant cations, with average concentrations of 2.6 μg/m^3^ and 2.2 μg/m^3^, respectively. Among anions, NO_3_^−^, Cl^−^, and SO_4_^2−^ were the major components, with mean concentrations of 5.5, 2.9, and 2.6 μg/m^3^, respectively. Cl^−^ and K^+^ are widely recognized as typical tracers of biomass burning (including residential wood combustion) [31]. NO_3_^−^ and SO_4_^2−^ are important constituents of secondary inorganic aerosols (SIA) [32], whereas Cl^−^ can also contribute to inorganic aerosol formation. All five ions exhibited pronounced seasonal variations, with wintertime average concentrations being 4.6–6.0 times higher than those in spring. This discrepancy can be attributed, on the one hand, to the sharp increase in residential biomass burning for heating purposes during winter, which likely resulted in substantially elevated emissions of primary tracers such as K^+^ and Cl^−^. On the other hand, the abundant volatile and semi-volatile organic compounds (e.g., phenols, carbonyl compounds) and gaseous inorganic precursors (e.g., NO_X_, HCl, SO_2_) released from wood combustion can be oxidized to form secondary inorganic ions such as NO_3_^−^ and SO_4_^2−^ via gas-phase oxidation or aqueous-phase reactions (in clouds, fog, or aerosol liquid water), particularly under stagnant and humid atmospheric conditions. Meanwhile, the organic precursors also contributed to the formation of aqueous secondary organic aerosol (aqSOA) [33,34]. Collectively, these processes elevated the concentrations of SIA and related components during winter.

### 3.2. Microbial Community Composition and Diversity

#### 3.2.1. Taxonomy Identification of Microbial Community

**Figure 2:**
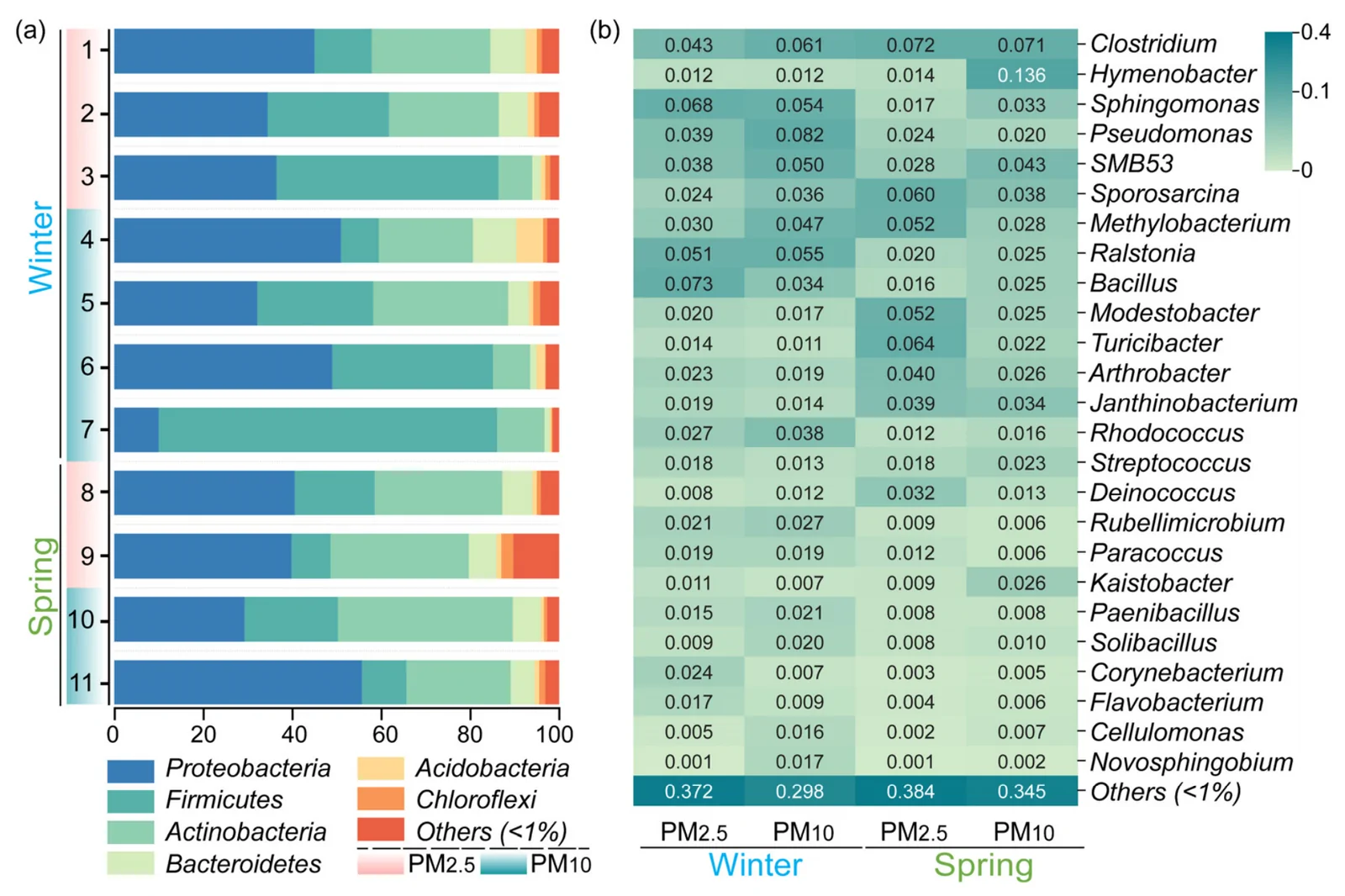
Seasonal variation in PM-associated bacterial communities: (**a**) relative abundances of bacterial phyla across seasons and particle-size fractions; (**b**) heatmap of the relative abundances of dominant bacterial genera across seasons and particle-size fractions.

In this study, a total of 375,935 high-quality 16S rRNA gene sequences and 761,211 high-quality 18S rRNA gene sequences were obtained. The relative abundances of bacterial communities at the phylum and genus levels are shown in Figure 2. Two-way ANOVA results for the dominant bacterial and eukaryotic genera are provided in Tables S11 and S12. At the phylum level (Figure 2a), *Proteobacteria*, *Firmicutes*, and *Actinobacteria* were the dominant phyla, with relative abundances of 38.5%, 26.8%, and 22.9%, respectively. The dominance of *Proteobacteria* is consistent with previous studies of airborne bacterial communities [35,36,37].

In terms of seasonal variation, the most pronounced change in community structure involved a replacement of *Firmicutes* by *Actinobacteria*. Specifically, the relative abundance of *Firmicutes* was high in winter (33.4%) but decreased substantially to 14.5% in spring. This winter enrichment of *Firmicutes* may have been driven by multiple factors. It is known that many members of *Firmicutes* (e.g., *Bacillus*) are capable of forming endospores, which confer strong resistance to environmental stress, allowing them to survive under harsh conditions such as low temperatures and desiccation. In addition, reduced precipitation and dry conditions in winter favor the resuspension of soil particles, thereby increasing the input of soil-associated microorganisms, such as *Bacillus* [38]. In contrast, the relative abundance of *Actinobacteria* increased from 18.7% in winter to 30.7% in spring. This phylum is widely distributed in soil and on plant surfaces, and its increase was likely associated with vegetation recovery and enhanced biological activity in spring, both of which may promote the release of biogenic aerosols [39]. Moreover, rising temperatures may enhance microbial metabolic activity and support the survival and dispersion of *Actinobacteria* in the atmosphere. Meanwhile, wind-driven resuspension processes can transport soil- and surface-associated microorganisms into the air. Studies from dust-affected regions have shown that atmospheric particles can act as carriers, facilitating the long-range transport of microorganisms [40].

Genus-level analysis (Figure 2b) further revealed that the dominant genera were *Clostridium*, *Hymenobacter*, and *Sphingomonas*, accounting for 6.2%, 4.3%, and 4.3%, respectively. *Clostridium* is a genus of Gram-positive, anaerobic, spore-forming bacteria widely distributed in natural environments such as soil, water, sewage, and the intestinal microbiota of humans and animals [41]. Some species within the genus *Clostridium* are known pathogens associated with gastrointestinal diseases and foodborne outbreaks [42]. Species within the genera *Sphingomonas* and *Hymenobacter* are known to tolerate environmental stresses such as desiccation, radiation, and oxidative stress and are frequently detected in nutrient-poor environments [43]. *Sphingomonas* is widely distributed on plant surfaces and is often considered a characteristic phyllosphere bacterium, suggesting that surrounding vegetation may contribute to its presence in the airborne community [44]. These findings suggest that the airborne bacterial community in Temuco was likely shaped by a combination of soil resuspension, vegetation-derived microorganisms, and atmospheric transport processes.

In winter, the relative abundance of *Pseudomonas* was higher than in spring. In PM_2.5_, its relative abundance reached 3.9%, which was approximately 1.6 times higher than in spring (2.4%). In PM_10_, the proportion increased to 8.2%, approximately 4.1 times the spring value (2.0%). *Bacillus* and *Corynebacterium* exhibited similar seasonal variation patterns. Previous studies have suggested that higher particulate matter concentrations and lower temperatures in winter may create favorable conditions for the survival and enrichment of certain airborne microorganisms, some of which may include opportunistic pathogens [1].

In spring, several soil-associated genera, including *Hymenobacter* (7.5%), *Turicibacter* (4.3%), and *Modestobacter* (3.8%), showed higher relative abundances. Spring is typically characterized by lower precipitation, higher wind speeds, and reduced vegetation cover, conditions that enhance the resuspension of surface particles and facilitate the emission of soil-associated bacteria into the atmosphere [45]. Under these conditions, typical soil-associated genera such as *Hymenobacter* and *Modestobacter* may be more easily transported into the air with dust particles. These genera generally exhibit strong resistance to radiation and desiccation and are capable of surviving under oligotrophic conditions, allowing them to remain detectable in particulate matter after soil–atmosphere transfer. Meanwhile, agricultural activities may have further contributed to the input of certain taxa. During the spring ploughing season, soil tillage and the application of organic fertilizers may disturb surface soils and increase the emission of gut-associated microorganisms, thereby contributing to the presence of genera such as *Turicibacter* in PM-associated airborne communities.

**Figure 3:**
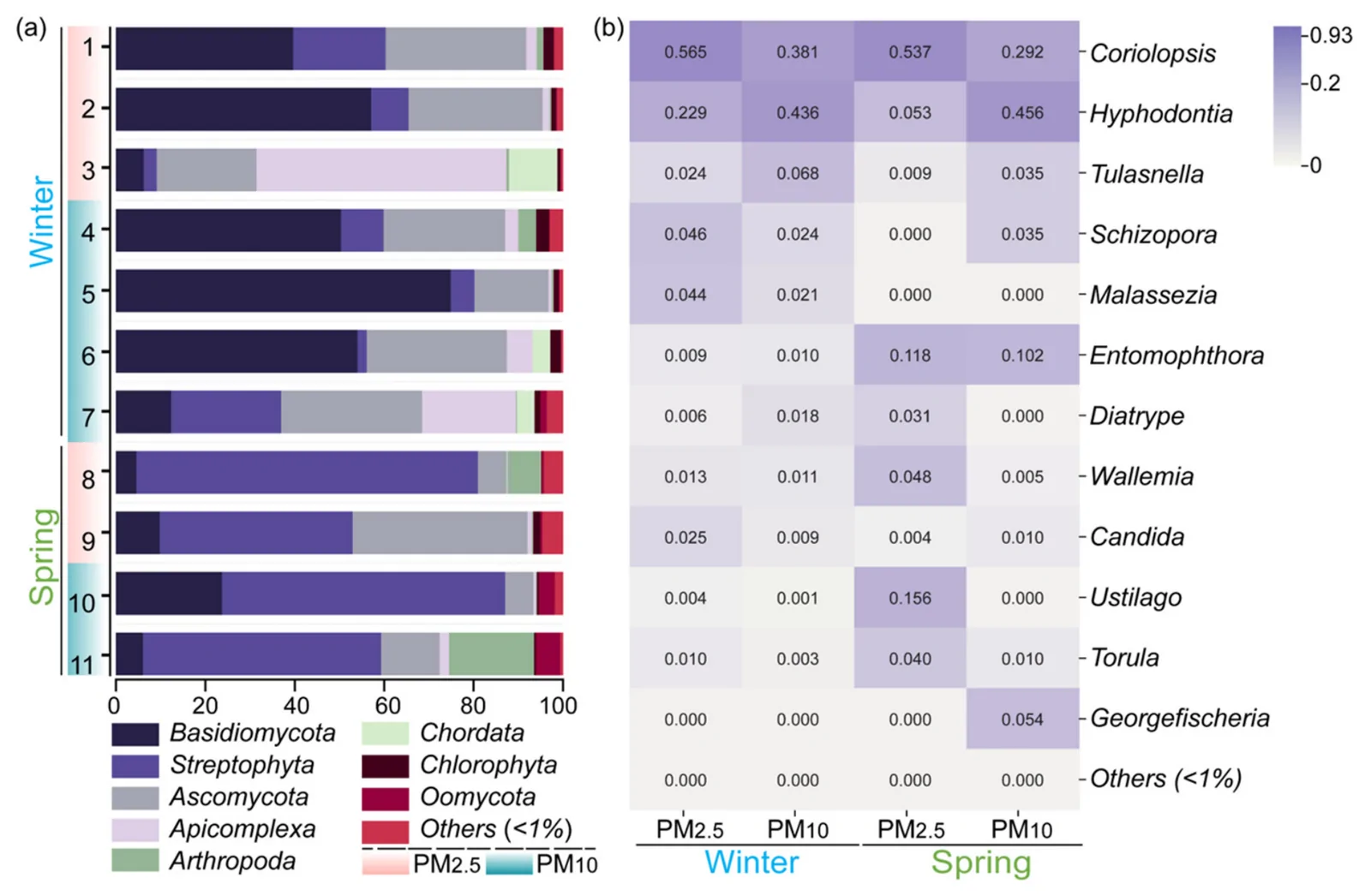
Seasonal variation in PM-associated eukaryotic communities: (**a**) relative abundances of eukaryotic phyla across seasons and particle-size fractions; (**b**) heatmap of the relative abundances of dominant eukaryotic genera across seasons and particle-size fractions.

In the eukaryotic community, Viridiplantae and Fungi were the dominant kingdoms, with *Streptophyta*, *Ascomycota*, and *Basidiomycota* as the major phyla [46]. At the phylum level (Figure 3a), *Basidiomycota*, *Streptophyta*, and *Ascomycota* were the dominant phyla, with mean relative abundances of 30.0%, 27.7%, and 22.7%, respectively. At the genus level (Figure 3b), *Coriolopsis* and *Hyphodontia* were the dominant genera, accounting for 41.0% and 26.9%, respectively. Both genera belong to the phylum *Basidiomycota*. Together, these two genera accounted for more than two-thirds of the total eukaryotic community. Among these, polypore fungi, including *Coriolopsis,* are perennial wood-decaying macrofungi with tough fruiting bodies, which can effectively reduce water loss under dry conditions. Moreover, their hyphae penetrate deep into host wood, allowing continuous access to moisture. This enables them to survive under alternating wet and dry conditions and may continuously release spores into the atmosphere [47]. Similarly, *Hyphodontia*, a typical corticioid basidiomycete, is primarily associated with decaying wood and bark substrates and is involved in lignocellulose decomposition [48].

#### 3.2.2. Diversity of Microbial Community

**Figure 4:**
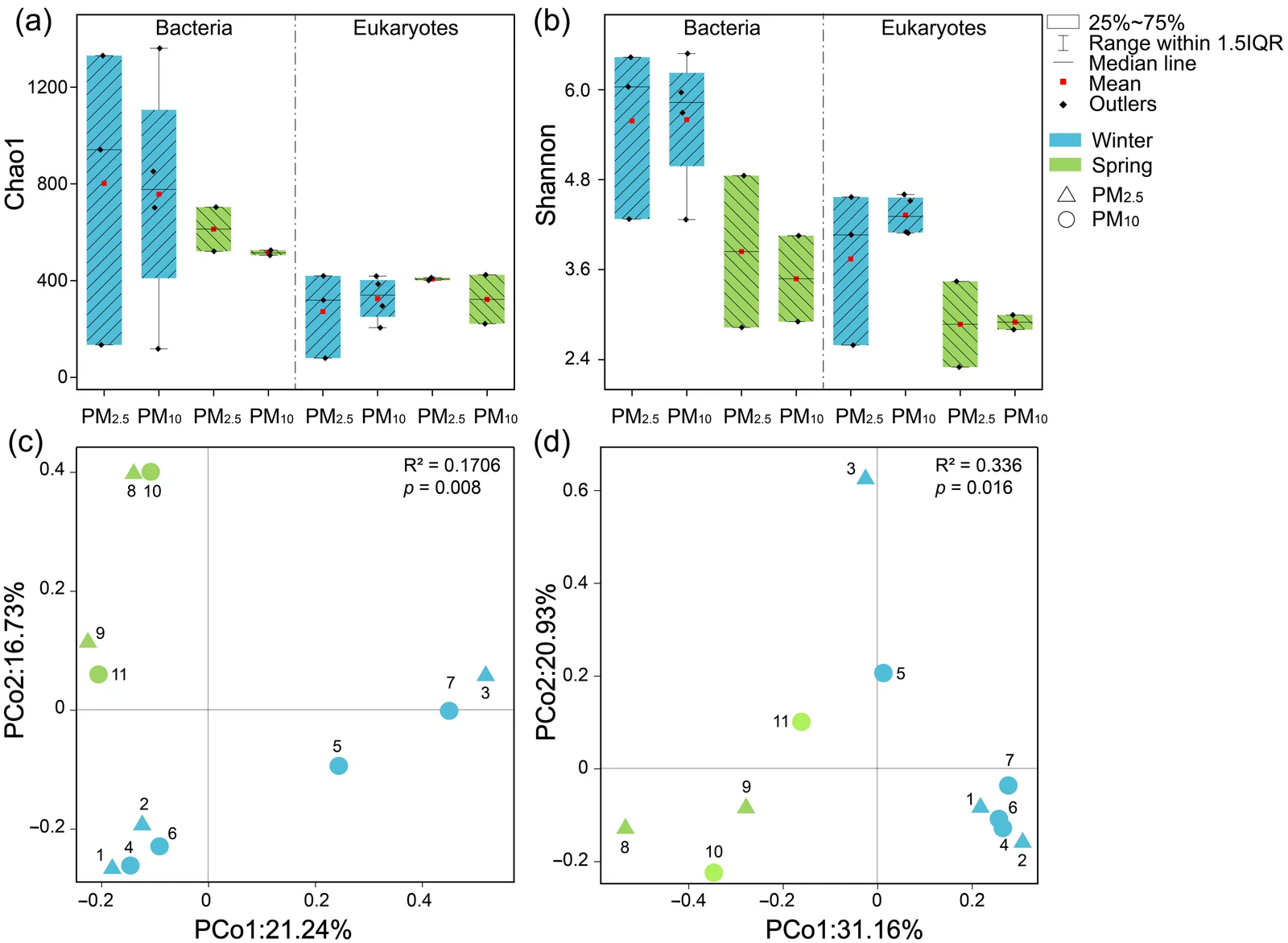
Alpha and Beta diversity of microbial communities across seasons and particle-size fractions. (**a**) Chao1 richness index; (**b**) Shannon diversity index; (**c**) Principal coordinate analysis (PCoA) of bacterial communities; and (**d**) PCoA of eukaryotic communities.

Chao1 and Shannon indices were used to assess microbial α-diversity. Chao1 estimates community richness, whereas the Shannon index reflects community diversity by incorporating both richness and evenness [49]. β-diversity was assessed using principal coordinate analysis (PCoA). As shown in Figure 4a,b, for bacterial communities, the mean Chao1 index values of winter PM_2.5_ and PM_10_ samples were 802.3 and 758.3, respectively, while the mean Shannon index values were both 5.6, higher than those observed in spring (mean Chao1 values of 613.0 and 515.5 and mean Shannon values of 3.8 and 3.5, respectively). The wider interquartile ranges and longer whiskers observed in winter also indicated greater variability among samples. These results suggest that bacterial communities associated with atmospheric PM during winter were characterized by higher species richness and microbial diversity than those in spring. The observed seasonal pattern may be associated with severe particulate pollution during winter, which is strongly influenced by residential wood combustion in Temuco. Under such conditions, low temperatures and reduced ultraviolet radiation may further decrease bacterial inactivation rates [50], thereby enhancing the atmospheric persistence of microorganisms originating from multiple sources. In addition, the lower atmospheric boundary layer height during winter may limit vertical dispersion and increase the residence time of PM and associated microorganisms near the surface [51,52]. Together, these factors may facilitate the accumulation and mixing of bacteria from diverse sources, contributing to the higher bacterial diversity and more heterogeneous community structure observed during winter.

In contrast to the pronounced seasonal differences observed for bacterial communities, the α-diversity of eukaryotic communities exhibited relatively limited seasonal variation in richness. The mean Chao1 index values ranged from 273.0 to 406.5 across seasons, suggesting that eukaryotic richness showed relatively limited seasonal variation. However, a clear seasonal pattern was observed for the Shannon index, with winter values (4.3 and 3.7 for PM_2.5_ and PM_10_, respectively) exceeding those recorded in spring (both 2.6), particularly for PM_10_ samples. The lower Shannon diversity of eukaryotic communities in spring may be related to the seasonal release of large quantities of pollen grains and fungal spores, which can temporarily increase the dominance of a limited number of taxa in the atmosphere [53]. Such seasonal shifts may increase the relative dominance of a limited number of taxa and consequently reduce Shannon diversity.

The PCoA results further revealed clear seasonal variations in community composition (Figure 4c,d). The cumulative variance explained by the first two principal coordinate axes was 38.0% for bacterial communities, which was lower than that for eukaryotic communities (52.1%). Nevertheless, both communities exhibited distinct seasonal separation patterns. Bacterial communities appeared more dispersed, whereas eukaryotic communities showed tighter clustering. Spring warming and vegetation recovery may enhance spore release and alter community composition, whereas winter conditions (low temperatures, high humidity, and biomass burning) may shift source contributions and reshape the community.

### 3.3. Correlations Between Microbial Communities and Environmental Factors

**Figure 5:**
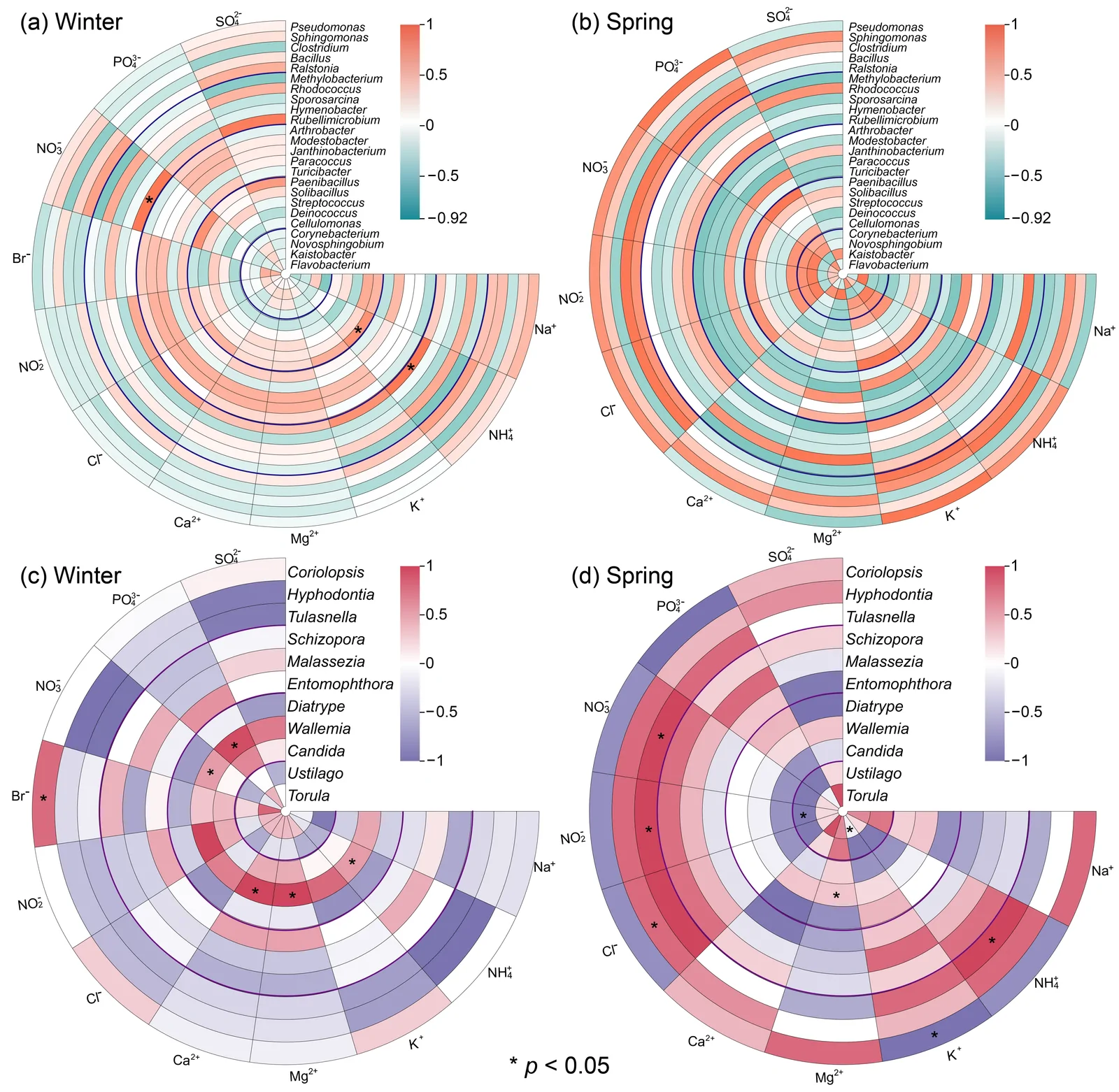
Relationship between dominant airborne microbial genera and WSIIs in Temuco. Colors represent correlation coefficients (r). Asterisks indicate significant correlations after Benjamini–Hochberg (BH) correction (BH-adjusted *p* < 0.05). (**a**) bacterial genera in winter; (**b**) bacterial genera in spring; (**c**) eukaryotic genera in winter; (**d**) eukaryotic genera in spring.

Correlation analysis revealed that NH_4_^+^ and NO_3_^−^ were the inorganic ions most strongly associated with bacterial genera in aerosols (Figure 5a), exhibiting similar correlation patterns. Specifically, both ions were significantly and positively correlated with *Rubellimicrobium* (r = 0.82 for each, BH-adjusted *p* < 0.05). In contrast, both ions were negatively correlated with *Methylobacterium* (r = −0.82 for both NH_4_^+^ and NO_3_^−^). *Rubellimicrobium*, which is primarily derived from soils and mineral surfaces [54], may reflect its tolerance to elevated ionic strength, potentially explaining its positive associations with these secondary inorganic ions. Conversely, *Methylobacterium* is a typical phyllosphere-associated genus that thrives on plant-derived methanol [43]. As the concentrations of secondary inorganic ions increase, the aerosol community may shift away from local biogenic sources, potentially resulting in a lower relative abundance of plant-affiliated taxa such as *Methylobacterium*.

In spring (Figure 5b), airborne bacterial communities showed correlations with Na^+^ and Mg^2+^. Notably, *Pseudomonas* exhibited a negative correlation with these ions (r = −0.8), which may reflect differences in source contributions. The Mg^2+^/Na^+^ ratio (0.137) was close to the seawater reference value (~0.12), suggesting a marine influence [55], whereas *Pseudomonas* is typically associated with local sources such as vegetation, aquatic environments, and moist soils. Therefore, its relative contribution may decrease under stronger marine air mass influence. In contrast, *Clostridium* showed a positive correlation with Na^+^ and Mg^2+^ (r = 0.8 for each), likely reflecting co-variation under marine-influenced conditions. As a spore-forming genus, *Clostridium* produces highly resistant spores capable of tolerating desiccation and salinity stress, potentially allowing them to persist during long-range atmospheric transport and increasing their likelihood of detection [56].

Among the eukaryotic genera (Figure 5c,d), in winter, *Wallemia* showed positive correlations with most WSIIs. *Wallemia* is a xerophilic fungus capable of growing under low water activity conditions and is commonly associated with food residues, dry organic matter, as well as bioaerosols and settled dust [57]. This genus exhibited a particularly strong positive correlation with Ca^2+^ and Mg^2+^ (r = 0.99, *p* < 0.05), suggesting a possible association with mineral dust particles or building materials enriched in these ions. This pattern may indicate that *Wallemia* can persist under conditions characterized by elevated concentrations of Ca^2+^ and Mg^2+^. In winter, low temperatures, reduced atmospheric mixing, and increased particle accumulation may contribute to simultaneous increases in soluble ions and airborne microbial concentrations. In spring, *Wallemia* showed a negative correlation with Ca^2+^ (r = −0.99). This may be explained by seasonal shifts in meteorological conditions and pollution sources: higher wind speeds may promote soil dust resuspension, increasing the concentrations of crustal ions such as Ca^2+^, while warmer temperatures and enhanced biogenic emissions from vegetation and soils may alter airborne microbial community structure, potentially reducing the relative dominance of xerophilic fungi.

**Figure 6:**
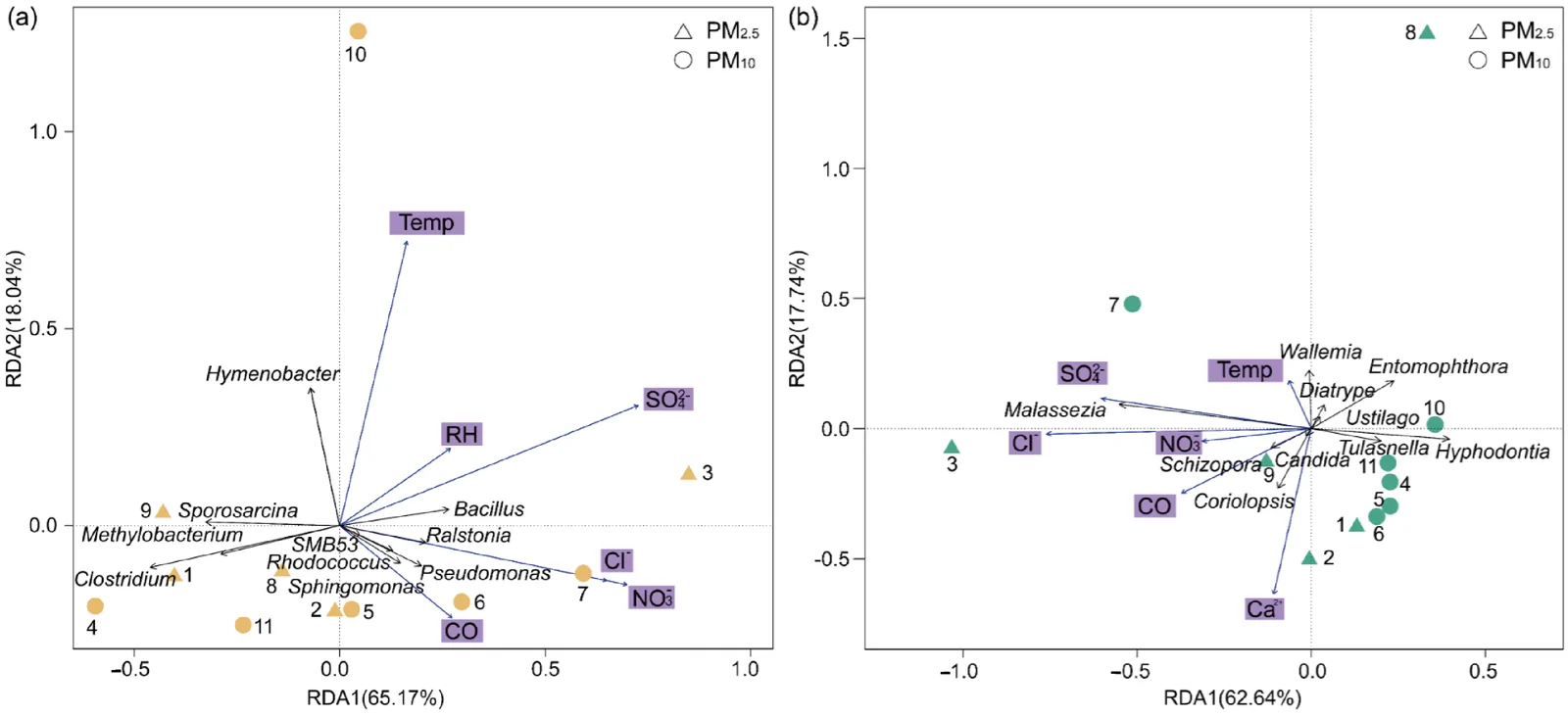
Redundancy analysis (RDA) of microbial communities in relation to environmental factors. (**a**) RDA of bacterial communities; (**b**) RDA of eukaryotic communities.

To minimize multicollinearity among environmental variables, variance inflation factor (VIF) screening was performed prior to RDA. Four rounds of screening for the bacterial dataset and five rounds for the eukaryotic dataset resulted in the retention of six environmental variables in each case (Tables S13 and S14). As shown in Figure 6 and Table 2, RDA revealed differences in the ordination patterns of bacterial and eukaryotic microbial communities in relation to the selected environmental variables, with the first two RDA axes explained 83.2% and 80.4% of the variation in bacterial and eukaryotic communities, respectively. For the bacterial community (Figure 6a), SO_4_^2−^ (loading = 0.7146) and NO_3_^−^ (loading = 0.6879) showed relatively high loadings on the RDA1 axis, suggesting an exploratory association between secondary inorganic aerosol components and bacterial community structure. Temp also contributed substantially to the RDA2 axis (loading = 0.7101), suggesting that seasonal meteorological conditions may further influence airborne bacterial community composition.

Further analysis of the relationships between environmental factors and major bacterial taxa showed that SO_4_^2−^ and NO_3_^−^ were oriented in similar directions to the arrows for Bacillus and Ralstonia, suggesting exploratory positive associations in the RDA ordination. Previous studies have similarly reported positive associations between ions such as SO_4_^2−^, NO_3_^−^, and NH_4_^+^ and Bacillus [58]. Bacillus can form spores, which confer high tolerance to polluted environments and atmospheric stress, potentially enhancing its survival under atmospheric stress and contributing to its persistence in environments with high levels of secondary inorganic aerosols. A relatively strong positive association was observed between Ralstonia and NO_3_^−^ (r = 0.78), which may be related to its nitrate-responsive capacity. Studies have shown that Ralstonia and its closely related Burkholderia groups possess the NarX–NarL nitrate-sensing system, which allows them to detect environmental NO_3_^−^ and regulate related metabolic and physiological processes [59]. Therefore, the similar directional orientation of Ralstonia and the NO_3_^−^ vector in this study may reflect an association between this bacterial group and nitrate-rich environments.

In the eukaryotic microbial community (Figure 6b), Cl^−^ exhibited the highest loading value (−0.9054) on the RDA1 axis and was positioned close to the third winter sample. This strong loading, together with the clustering of winter samples, suggests an exploratory association between the Cl^−^ gradients and seasonal variation in eukaryotic community structure, consistent with previous reports that wintertime combustion emissions significantly alter fungal assemblages [60]. SO_4_^2−^ had a loading of −0.7176 on the RDA1 axis and was very close to the vector of *Malassezia*. *Malassezia* is a common lipophilic yeast associated with indoor air environments and is widely distributed on human skin and in the atmosphere. This pattern may reflect an exploratory association between sulfate-containing aerosol particles and *Malassezia*. The strong hygroscopicity of sulfate-containing particles could potentially provide favorable conditions for the attachment and persistence of airborne fungi, although this interpretation requires further investigation. Despite these findings, several methodological limitations should be acknowledged. The relatively small sample size may increase the risk of spurious correlations in correlation analyses and reduce the robustness of RDA ordination. Therefore, these findings should ideally be validated using larger datasets in future studies.

## 4. Conclusions

This study examined the distribution of bacterial and eukaryotic communities in atmospheric aerosols of different particle sizes and seasons in Temuco, a city dominated by residential wood combustion in southern Chile. Mean PM_2.5_ and PM_10_ concentrations were 75.0 and 87.7 μg/m^3^, respectively, with PM_2.5_ exceeding the Chilean national air quality standard. Among the water-soluble inorganic ions (WSIIs), the secondary inorganic ions NO_3_^−^ (5.6 μg/m^3^) and SO_4_^2−^ (2.6 μg/m^3^), together with the biomass burning tracer K^+^ (2.6 μg/m^3^), were the most abundant ions. Bacterial communities exhibited higher richness and diversity in winter, whereas eukaryotic communities showed limited seasonal variation in richness but clear differences in Shannon diversity. Winter conditions were strongly influenced by residential wood combustion, resulting in higher PM concentrations and elevated levels of combustion-related ions. These findings suggest that winter biomass burning not only contributes primary particulate matter but may also exacerbate fine-particle pollution through secondary aerosol formation processes. Redundancy analysis (RDA) showed relatively high loadings for SO_4_^2−^ (loading = 0.7146) and temperature (Temp, loading = 0.7101) in the bacterial RDA, whereas Cl^−^ (loading = −0.9054) and SO_4_^2−^ (loading = −0.7176) showed relatively high loadings in the eukaryotic RDA. Overall, this study provides baseline information on airborne microbial communities and their potential environmental associations in a city dominated by residential wood combustion. Reducing residential wood combustion, particularly during winter, may help mitigate PM_2.5_ pollution and improve air quality in Temuco.

## Figures and Tables

**Table 1:** Sampling information.

Season	Order	Sampling Date	Fraction	Temp (°C)	RH (%)	Wind Speed (m/s)	PM_2.5_ (μg/m^3^)	PM_10_ (μg/m^3^)
Winter	1	15 June 2021	PM_2.5_	7.2	66.5	1.1	66	78
	2	17 June 2021	PM_2.5_	13.9	86.7	0.6	153	173
	3	30 June 2021	PM_2.5_	7.8	95.3	1	117	125
	4	14 June 2021	PM_10_	4.5	84.7	1.4	87	90
	5	16 June 2021	PM_10_	15.9	84.3	0.8	140	164
	6	23 June 2021	PM_10_	10.3	82.5	2.4	12	15
	7	29 June 2021	PM_10_	5.3	91.2	0.6	157	182
Spring	8	7 September 2021	PM_2.5_	9.6	75.7	1.1	28	36
	9	13 October 2021	PM_2.5_	4.6	81.4	2.8	11	45
	10	8 September 2021	PM_10_	12.9	87.9	1.4	36	42
	11	12 October 2021	PM_10_	16	86.5	1	18	15

**Table 2:** Ranked explanatory variables and eigenvalues from RDA.

Bacterial Community			Eukaryotic Community		
Factors	RDA1 (65.17%)	RDA2 (18.04%)	Factors	RDA1 (62.64%)	RDA2 (17.74%)
	Explanatory Plot	Explanatory Plot		Explanatory Plot	Explanatory Plot
Cl^−^	0.6390	−0.1374	Ca^2+^	−0.1280	−0.7564
SO_4_^2−^	0.7146	0.3002	Cl^−^	−0.9054	−0.0246
Temp	0.1609	0.7101	NO_3_^−^	−0.3735	−0.0582
CO	0.2690	−0.2307	SO_4_^2−^	−0.7176	0.1378
RH	0.2650	0.1930	Temp	−0.0757	0.2194
NO_3_^−^	0.6879	−0.1479	CO	−0.4428	−0.2963

## Data Availability

Data will be made available on request. Additional data supporting the findings of this study are available from the corresponding author upon reasonable request.

## Supplementary Materials

The following supporting information can be downloaded at https://www.mdpi.com/article/10.3390/life16091433/s1: Table S1: Sequencing statistics of bacterial samples and field blanks. Table S2: Sequencing statistics of eukaryotic samples and field blanks. Table S3: Benjamini–Hochberg adjusted *p* values for correlations between environmental variables and bacteria communities in Winter. Table S4: Benjamini–Hochberg adjusted *p* values for correlations between environmental variables and bacteria communities in Spring. Table S5: Benjamini–Hochberg adjusted *p* values for correlations between environmental variables and eukaryotic communities in Winter. Table S6: Benjamini–Hochberg adjusted *p* values for correlations between environmental variables and eukaryotic communities in Spring. Table S7: Permutation test for overall RDA model and canonical axes. Table S8: Significance test of environmental variables in RDA using permutation tests. Table S9: Permutation test for overall RDA model and canonical axes. Table S10: Significance test of environmental variables in RDA using permutation tests. Table S11: Two-way ANOVA results showing the effects of season and particle-size fraction on the relative abundances of dominant bacteria genera. Table S12: Two-way ANOVA results showing the effects of season and particle-size fraction on the relative abundances of dominant eukaryotic genera. Table S13: Variance inflation factor (VIF) values of environmental variables during the stepwise variable selection process prior to redundancy analysis (RDA) of bacteria communities. Table S14: VIF values of environmental variables during the stepwise variable selection process prior to RDA of eukaryotic communities.

## Abbreviations

The following abbreviations are used in this manuscript:

RH	Relative humidity
WSIIs	Water-soluble inorganic ions
