# Supplementary material for: Airborne Bacterial and Eukaryotic Communities in Temuco, Chile, a City Dominated by Residential Wood Combustion
Source: Life (Basel). 2026 Aug 28;16(9):1433. doi: 10.3390/life16091433 (PMC13608280; doi:10.3390/life16091433)
Supplement: Supplementary file 1 [file life-16-01433-s001.zip › life-4451133-supplementary.pdf]

Supplementary Materials

Airborne Bacterial and Eukaryotic Communities in Temuco, Chile, a City Dominated by Residential Wood Combustion

Juan Liu [1], So Fujiyoshi [2,3], Fumito Maruyama [2], Jun Noda [4], Tay Ruiz-Gil [5,6], Elizabeth Carrazana [5,7], Jacquelinne J. Acuña [5], Milko A. Jorquera [5], Nobutake Nakatani [8], Shiyuan Guo [1], Akihiro Sakatoku [1] and Daisuke Tanaka [1,*]

[1] Graduate School of Science and Engineering, University of Toyama, Toyama, Japan.
[2] Microbial Genomics and Ecology, The IDEC Institute, Hiroshima University, Hiroshima, Japan.
[3] Department of Environmental and Civil Engineering, Faculty of Engineering, Toyama Prefectural University, Toyama, Japan.
[4] Graduate School of Veterinary Medicine, Rakuno Gakuen University, Hokkaido, Japan.
[5] Laboratorio de Ecología Microbiana Aplicada, Departamento de Ciencias Químicas y Recursos Naturales, Universidad de La Frontera, Temuco, Chile.
[6] Centro de Investigación, Innovación y Creación, Vicerrectoría de Investigación y Posgrado, Universidad Católica de Temuco, Temuco, Chile.
[7] Laboratory of Neurodegenerative Diseases, Center for Biomedicine, Universidad Mayor, Temuco, Chile.
[8] Department of Environmental Sciences, College of Agriculture, Food and Environment Sciences, Rakuno Gakuen University, Hokkaido, Japan.
* Correspondence: tanakada@sci.u-toyama.ac.jp; Tel.: +81-76-445-6673

Table S1	Sequencing statistics of bacterial samples and field blanks.
Table S2	Sequencing statistics of eukaryotic samples and field blanks.
Table S3	Benjamini–Hochberg adjusted *p* values for correlations between environmental variables and bacteria communities in Winter.
Table S4	Benjamini–Hochberg adjusted *p* values for correlations between environmental variables and bacteria communities in Spring.
Table S5	Benjamini–Hochberg adjusted *p* values for correlations between environmental variables and eukaryotic communities in Winter.
Table S6	Benjamini–Hochberg adjusted *p* values for correlations between environmental variables and eukaryotic communities in Spring.
Table S7	Permutation test for overall RDA model and canonical axes.
Table S8	Significance test of environmental variables in RDA using permutation tests.
Table S9	Permutation test for overall RDA model and canonical axes.
Table S10	Significance test of environmental variables in RDA using permutation tests.
Table S11	Two-way ANOVA results showing the effects of season and particle-size fraction on the relative abundances of dominant bacteria genera.
Table S12	Two-way ANOVA results showing the effects of season and particle-size fraction on the relative abundances of dominant eukaryotic genera.
Table S13	Variance inflation factor (VIF) values of environmental variables during the stepwise variable selection process prior to redundancy analysis (RDA) of bacteria communities.
Table S14	VIF values of environmental variables during the stepwise variable selection process prior to RDA of eukaryotic communities.

Table S1. Sequencing statistics of bacterial samples and field blanks.

No.	Sample name		Library concentration measurement results		Sequencing results			Filtering results	Microbial ASVs
			Concentration (ng/μL)	Volume (μL)	Paired-end reads	Q20 (%)	Q30 (%)	Merged sequences	
1	2021/6/15	$PM_{2.5}$	20.0	20	76,988	88.4	77.3	40,422	34,488
2	2021/6/17	$PM_{2.5}$	20.0	20	75,486	88.0	76.9	41,074	38,395
3	2021/6/30	$PM_{2.5}$	14.8	20	71,135	88.9	78.1	35,605	34,779
4	2021/6/14	PM_{10}	17.5	20	79,592	88.2	77.2	40,980	36,486
5	2021/6/16	PM_{10}	21.3	20	78,867	88.2	77.1	41,960	38,146
6	2021/6/23	PM_{10}	21.8	20	86,170	88.8	77.9	46,136	40,955
7	2021/6/29	PM_{10}	24.3	20	75,741	88.5	77.4	42,993	41,320
8	2021/9/7	$PM_{2.5}$	22.3	20	72,389	88.3	77.5	49,091	10,892
9	2021/10/13	$PM_{2.5}$	18.4	20	76,129	88.1	77.2	44,273	23,109
10	2021/9/8	PM_{10}	23.1	20	86,762	88.1	77.3	55,884	13,338
11	2021/10/12	PM_{10}	24.2	20	91,899	89.2	78.5	48,171	20,717
	Blank1		0.168	20	6,268	88.7	77.8	4,479	4,479
	Blank2		0.724	20	26,430	88.3	77.0	18,358	18,358
	Blank3		3.85	20	41,519	88.9	78.0	29,204	28,525
	Blank4		0.310	20	12,516	88.8	77.7	8,699	8,699

Table S2. Sequencing statistics of eukaryotic samples and field blanks.

No.	Sample name		Library concentration measurement results		Sequencing results			Filtering results	Microbial ASVs
			Concentration (ng/μL)	Volume (μL)	Paired-end reads	Q20 (%)	Q30 (%)	Merged sequences	
1	2021/6/15	$PM_{2.5}$	12.8	20	76,824	94.3	89.0	63,913	63,226
2	2021/6/17	$PM_{2.5}$	12.6	20	81,250	93.6	88.1	67,385	66,938
3	2021/6/30	$PM_{2.5}$	13.5	20	81,951	95.2	90.6	71,338	71,323
4	2021/6/14	PM_{10}	15.1	20	79,487	93.6	88.0	67,201	66,472
5	2021/6/16	PM_{10}	12.9	20	79,003	93.8	88.3	66,943	66,255
6	2021/6/23	PM_{10}	13.9	20	86,370	95.0	90.1	67,269	67,061
7	2021/6/29	PM_{10}	15.9	20	97,731	94.8	89.9	79,907	79,726
8	2021/9/7	$PM_{2.5}$	11.6	20	78,456	95.3	90.4	69,590	68,162
9	2021/10/13	$PM_{2.5}$	10.3	20	80,514	94.9	90.2	70,401	69,264
10	2021/9/8	PM_{10}	10.5	20	79,379	94.8	89.8	70,505	69,301
11	2021/10/12	PM_{10}	13.9	20	84,538	94.1	88.3	73,953	73,483
	Blank1		Low	20	3,202	92.9	87.0	2,829	2,829
	Blank2		Low	20	6,525	92.5	86.5	5,820	5,820
	Blank3		0.102	20	7,147	92.9	87.0	6,143	6,123
	Blank4		Low	20	5,131	92.5	86.6	4,420	4,420

Table S3. Benjamini–Hochberg adjusted *p* values for correlations between environmental variables and bacteria communities in Winter.

p values	Na^+	NH_4^+	K^+	Mg^{2+}	Ca^{2+}	Cl^-	NO_2^-	Br^-	NO_3^-	PO_4^{3-}	SO_4^{2-}
Pseudomonas	0.8392	0.8438	0.9760	0.8576	0.8950	0.9760	0.9760	0.8438	0.8438	0.8865	0.9809
Sphingomonas	0.9760	0.9390	0.8438	0.8438	0.8438	0.8438	0.8438	0.8438	0.9349	0.8438	0.8865
Clostridium	0.8438	0.8438	0.8438	0.8438	0.8438	0.8438	0.8516	0.9509	0.8438	0.8576	0.6795
Bacillus	0.8950	0.9847	0.8576	0.8438	0.8438	0.8576	0.8576	0.8438	0.9760	0.8438	0.9809
Ralstonia	0.8727	0.3862	0.8438	0.8438	0.8438	0.8950	0.8438	0.8438	0.4965	0.8438	0.7387
Methylobacterium	0.8576	0.6743	0.8438	0.8438	0.8438	0.8438	0.8438	0.9760	0.8438	0.8438	0.3862
Rhodococcus	0.8393	0.1660	0.8438	0.5646	0.6665	0.8576	0.8438	0.8576	0.1660	0.5103	0.8392
Sporosarcina	0.8438	0.8438	0.8438	0.8727	0.8727	0.8438	0.9349	0.9760	0.8438	0.9269	0.8438
Hymenobacter	0.9238	0.9349	0.8438	0.8576	0.8438	0.8438	0.8438	0.8576	0.9430	0.8438	0.8576
Rubellimicrobium	0.8438	0.0022	0.8438	0.3862	0.3862	0.8438	0.3227	0.9179	0.0183	0.2632	0.3862
Arthrobacter	0.8576	0.8438	0.9349	0.9760	0.9921	0.9179	0.9760	0.8438	0.8438	0.9760	0.9430
Modestobacter	0.9349	0.9219	0.8438	0.9179	0.8865	0.7387	0.8950	0.8438	0.8640	0.9760	0.8865
Janthinobacterium	0.9809	0.8634	0.8438	0.9760	0.9349	0.8438	0.9349	0.8438	0.8438	0.9809	0.9430
Paracoccus	0.9430	0.9760	0.8438	0.8438	0.8438	0.8438	0.8438	0.8438	0.9760	0.8438	0.8865
Turicibacter	0.8575	0.8727	0.9411	0.9809	0.9641	0.9349	0.9430	0.8438	0.8619	0.9760	0.9847
Paenibacillus	0.8438	0.0098	0.8438	0.3862	0.3862	0.8438	0.3862	0.8576	0.0710	0.3862	0.3862
Solibacillus	0.6960	0.3227	0.8438	0.3862	0.6108	0.8576	0.8438	0.8438	0.2632	0.5318	0.8438
Streptococcus	0.8438	0.8438	0.9760	0.9430	0.9760	0.9760	0.9760	0.8438	0.8438	0.9569	0.9430

Table S3. (continued)

p values	Na^{+}	NH_4^{+}	K^{+}	Mg^{2+}	Ca^{2+}	Cl^{-}	NO_2^{-}	Br^{-}	NO_3^{-}	PO_4^{3-}	SO_4^{2-}
Deinococcus	0.8438	0.8438	0.9179	0.9219	0.8865	0.9921	0.8438	0.8727	0.8438	0.8438	0.8438
Cellulomonas	0.8950	0.8576	0.8438	0.8438	0.8438	0.8438	0.6665	0.8438	0.8865	0.8438	0.8438
Corynebacterium	0.6960	0.8551	0.8438	0.8576	0.8950	0.8438	0.9760	0.8438	0.8727	0.9301	0.8865
Novosphingobium	0.8576	0.8461	0.8438	0.8640	0.8438	0.8438	0.6795	0.8727	0.8865	0.8516	0.8392
Kaistobacter	0.8865	0.8438	0.8933	0.9760	0.9923	0.8576	0.9760	0.8438	0.8438	0.9430	0.9809
Flavobacterium	0.8438	0.8438	0.9760	0.8576	0.9025	0.9760	0.9809	0.8438	0.8438	0.8950	0.9430

Table S4. Benjamini–Hochberg adjusted *p* values for correlations between environmental variables and bacteria communities in Spring.

p values	Na^+	NH_4^+	K^+	Mg^{2+}	Ca^{2+}	Cl^-	NO_2^-	NO_3^-	PO_4^{3-}	SO_4^{2-}
Pseudomonas	0.7227	0.7481	0.6479	0.6482	0.7662	0.8315	0.7662	0.7710	0.6479	0.9052
Sphingomonas	0.8644	0.8574	0.9497	0.9497	0.9052	0.9052	0.7521	0.7662	0.9748	0.6609
Clostridium	0.7662	0.7706	0.7597	0.9052	0.9306	0.7521	0.9052	0.8498	0.8851	0.8954
Bacillus	0.8172	0.6482	0.6482	0.9052	0.9748	0.6479	0.7662	0.7227	0.8182	0.9814
Ralstonia	0.8172	0.6479	0.6479	0.7889	0.9052	0.7481	0.6482	0.6482	0.6998	0.9748
Methylobacterium	0.7481	0.9762	0.9052	0.6482	0.6479	0.9052	0.9206	0.9052	0.7662	0.6998
Rhodococcus	0.6479	0.9087	0.7710	0.7597	0.7521	0.9283	0.9806	0.9884	0.7662	0.6482
Sporosarcina	0.7710	0.9052	0.9982	0.7662	0.6482	0.7889	0.8172	0.7889	0.8954	0.6482
Hymenobacter	0.9497	0.7662	0.8182	0.9497	0.9052	0.6482	0.8386	0.7662	0.9814	0.9497
Rubellimicrobium	0.6479	0.8197	0.7227	0.6482	0.6998	0.9052	0.9306	0.9093	0.6609	0.7597
Arthrobacter	0.8315	0.6479	0.6479	0.8197	0.9206	0.6998	0.6479	0.6479	0.7481	0.9558
Modestobacter	0.9748	0.6479	0.7662	0.9814	0.9296	0.6479	0.6479	0.6479	0.9010	0.8211
Janthinobacterium	0.8386	0.6479	0.6479	0.8182	0.9087	0.7481	0.6479	0.6479	0.7481	0.9497
Paracoccus	0.6482	0.7662	0.6482	0.6479	0.6998	0.9052	0.8574	0.8644	0.6479	0.8197
Turicibacter	0.8985	0.7662	0.9052	0.9052	0.7936	0.7662	0.6971	0.6998	0.9965	0.6482
Paenibacillus	0.7597	0.6482	0.6479	0.7481	0.7917	0.7662	0.7521	0.7521	0.6479	0.9497
Solibacillus	0.9087	0.9052	0.9497	0.9748	0.9816	0.9806	0.7662	0.8386	0.9087	0.7662
Streptococcus	0.7662	0.9052	0.8182	0.9087	0.9283	0.8172	0.9995	0.9497	0.9107	0.7917

Table S4. Continued

p values	Na^+	NH_4^+	K^+	Mg^{2+}	Ca^{2+}	Cl^-	NO_2^-	NO_3^-	PO_4^{3-}	SO_4^{2-}
Deinococcus	0.9825	0.7227	0.7889	0.9558	0.9654	0.7662	0.6479	0.6482	0.8574	0.7662
Cellulomonas	0.8954	0.7481	0.7597	0.9748	0.9748	0.6479	0.7917	0.7597	0.9052	0.9995
Corynebacterium	0.7662	0.6482	0.6482	0.8740	0.9296	0.6482	0.7662	0.7521	0.7723	0.9497
Novosphingobium	0.7748	0.6482	0.6482	0.9052	0.9497	0.6479	0.7662	0.7481	0.7889	0.9654
Kaistobacter	0.6479	0.9052	0.7662	0.7597	0.7597	0.9087	0.9825	0.9814	0.7662	0.6609
Flavobacterium	0.9306	0.7662	0.7748	0.8197	0.9052	0.9052	0.7227	0.7662	0.7662	0.9052

Table S5. Benjamini–Hochberg adjusted *p* values for correlations between environmental variables and eukaryotic communities in Winter.

p values	Na^+	NH_4^+	K^+	Mg^{2+}	Ca^{2+}	Cl^-	NO_2^-	Br^-	NO_3^-	PO_4^{3-}	SO_4^{2-}
Coriolopsis	0.9943	0.9943	0.8170	0.9943	0.9943	0.8170	0.9943	0.0809	0.9943	0.9943	0.9943
Hyphodontia	0.9943	0.8170	0.9344	0.9943	0.9943	0.9943	0.8299	0.9943	0.8999	0.9943	0.8170
Tulasnella	0.9943	0.8170	0.8170	0.9249	0.8251	0.8170	0.6269	0.9943	0.8170	0.8170	0.6269
Schizopora	0.8170	0.9943	0.9943	0.8170	0.8170	0.9943	0.9943	0.9943	0.9943	0.8170	0.9943
Malassezia	0.9943	0.8170	0.9943	0.9943	0.9943	0.9943	0.9688	0.8999	0.8170	0.9943	0.8170
Entomophthora	0.9943	0.9344	0.9943	0.8170	0.8170	0.9943	0.8170	0.9943	0.8170	0.8170	0.9943
Diatrype	0.9943	0.9943	0.8170	0.9943	0.9943	0.8170	0.9943	0.8921	0.9943	0.9943	0.7548
Wallemia	0.8170	0.0367	0.6597	0.0035	0.0057	0.8170	0.0927	0.9943	0.0367	0.0035	0.6582
Candida	0.9943	0.9943	0.9943	0.9344	0.8999	0.9943	0.8251	0.9943	0.8921	0.8170	0.9943
Ustilago	0.8170	0.8999	0.8921	0.9943	0.9943	0.8170	0.9943	0.9943	0.9943	0.9943	0.8999
Torula	0.9943	0.9943	0.8170	0.9943	0.9943	0.8170	0.9943	0.0809	0.9943	0.9943	0.9943

Table S6. Benjamini–Hochberg adjusted *p* values for correlations between environmental variables and eukaryotic communities in Spring.

p values	Na^+	NH_4^+	K^+	Mg^{2+}	Ca^{2+}	Cl^-	NO_2^-	NO_3^-	PO_4^{3-}	SO_4^{2-}
Coriolopsis	0.7865	0.6010	0.5263	0.7471	0.8450	0.7865	0.6902	0.7169	0.5263	0.9771
Hyphodontia	0.9982	0.6902	0.8290	0.9771	0.8761	0.5263	0.7654	0.6844	0.9771	0.8761
Tulasnella	0.9771	0.5263	0.6902	0.9771	0.9771	0.5263	0.5263	0.0995	0.8705	0.8761
Schizopora	0.9771	0.7865	0.8521	0.9771	0.9174	0.5263	0.8705	0.7865	0.9982	0.9771
Entomophthora	0.7865	0.8761	0.7865	0.6010	0.6902	0.9982	0.8761	0.9399	0.5263	0.9753
Diatrype	0.7856	0.9771	0.9771	0.6902	0.5263	0.8761	0.9174	0.8761	0.8290	0.6844
Wallemia	0.6902	0.9399	0.7865	0.5214	0.5263	0.9982	0.9771	0.9771	0.5263	0.8290
Candida	0.8761	0.9982	0.9771	0.9982	0.9771	0.9771	0.9174	0.9771	0.9982	0.7865
Ustilago	0.9771	0.6844	0.7865	0.9771	0.9982	0.7865	0.5214	0.5263	0.8290	0.8548
Torula	0.7865	0.5263	0.0995	0.7865	0.8761	0.6902	0.7278	0.6902	0.6902	0.9771
Georgefischeria	0.7865	0.8761	0.9982	0.7865	0.6902	0.8393	0.7865	0.7865	0.9399	0.5263

Table S7. Permutation test for overall RDA model and canonical axes.

Term	Df	Variance	F value	*p* value
Model	6	0.1268	1.3652	0.3000
RDA1	1	0.0826	5.3387	0.3400
RDA2	1	0.0229	1.8472	0.7730
RDA3	1	0.0127	1.2277	0.8980
RDA4	1	0.0064	0.7183	0.9520
RDA5	1	0.0013	0.1648	0.9980
RDA6	1	0.0010	0.1432	0.9980

Table S8. Significance test of environmental variables in RDA using permutation tests.

Term	Df	Variance	F value	*p* value
Cl^-	1	0.0372	2.4013	0.1070
SO_4^{2-}	1	0.0238	1.5351	0.2190
Temp	1	0.0152	0.9813	0.3820
CO	1	0.0073	0.4684	0.7360
RH	1	0.0047	0.3014	0.8520
NO_3^-	1	0.0388	2.5040	0.1000

Table S9. Permutation test for overall RDA model and canonical axes.

Term	Df	Variance	F value	*p* value
Overall model	6	0.1489	0.9461	0.5330
RDA1	1	0.0933	3.5559	0.3930
RDA2	1	0.0264	1.2592	0.9290
RDA3	1	0.0151	0.8631	0.9680
RDA4	1	0.0087	0.5824	0.9930
RDA5	1	0.0035	0.2658	0.9970
RDA6	1	0.0019	0.1623	0.9970

Table S10. Significance test of environmental variables in RDA using permutation tests.

Term	Df	Variance	F value	*p* value
Ca^{2+}	1	0.0190	0.7245	0.5820
Cl^{-}	1	0.0769	2.9310	0.042*
NO_3^{-}	1	0.0073	0.2786	0.9410
SO_4^{2-}	1	0.0077	0.2942	0.9500
Temp máx	1	0.0145	0.5513	0.6390
CO	1	0.0235	0.8968	0.5340

Table S11. Two-way ANOVA results showing the effects of season and particle-size fraction on the relative abundances of dominant bacteria genera.

Genus	Winter mean	Spring mean	$PM_{2.5}$ mean	PM_{10} mean	Season *p*	Size *p*	Season sig	Size sig
Pseudomonas	0.0636	0.0218	0.0326	0.0616	0.1618	0.3455	ns	ns
Sphingomonas	0.0599	0.0253	0.0474	0.0472	0.4028	0.9484	ns	ns
Clostridium	0.0533	0.0719	0.0549	0.0644	0.5599	0.7250	ns	ns
Bacillus	0.0509	0.0207	0.0503	0.0312	0.4731	0.6006	ns	ns
Ralstonia	0.0533	0.0229	0.0387	0.0452	0.3101	0.8724	ns	ns
SMB53	0.0451	0.0356	0.0340	0.0480	0.6699	0.5400	ns	ns
Methylobacterium	0.0399	0.0399	0.0388	0.0408	0.9972	0.9079	ns	ns
Rhodococcus	0.0335	0.0143	0.0209	0.0311	0.2170	0.5372	ns	ns
Sporosarcina	0.0313	0.0491	0.0387	0.0370	0.3935	0.9774	ns	ns
Hymenobacter	0.0118	0.0747	0.0125	0.0531	0.2025	0.3344	ns	ns
Rubellimicrobium	0.0248	0.0073	0.0162	0.0202	0.2621	0.8422	ns	ns
Arthrobacter	0.0209	0.0332	0.0297	0.0217	0.3655	0.5801	ns	ns
Modestobacter	0.0183	0.0384	0.0328	0.0197	0.1276	0.3331	ns	ns
Janthinobacterium	0.0164	0.0369	0.0273	0.0210	0.0463	0.5738	*	ns
Paracoccus	0.0191	0.0090	0.0161	0.0149	0.3841	0.8662	ns	ns
Turicibacter	0.0123	0.0428	0.0338	0.0146	0.0180	0.1213	*	ns
Paenibacillus	0.0183	0.0077	0.0118	0.0166	0.3116	0.6748	ns	ns
Solibacillus	0.0156	0.0087	0.0087	0.0167	0.2277	0.1749	ns	ns
Streptococcus	0.0152	0.0205	0.0180	0.0164	0.6129	0.9027	ns	ns
Deinococcus	0.0101	0.0227	0.0179	0.0120	0.1244	0.4965	ns	ns
Cellulomonas	0.0113	0.0045	0.0038	0.0130	0.5799	0.4620	ns	ns
Corynebacterium	0.0142	0.0041	0.0155	0.0063	0.3713	0.3656	ns	ns
Novosphingobium	0.0103	0.0014	0.0008	0.0122	0.4966	0.3945	ns	ns
Kaistobacter	0.0088	0.0174	0.0101	0.0134	0.3141	0.6295	ns	ns
Flavobacterium	0.0123	0.0050	0.0119	0.0079	0.1770	0.3738	ns	ns
Others	0.3297	0.3644	0.3768	0.3136	0.6620	0.4317	ns	ns

Table S12. Two-way ANOVA results showing the effects of season and particle-size fraction on the relative abundances of dominant eukaryotic genera.

Genus	Winter mean	Spring mean	$PM_{2.5}$ mean	PM_{10} mean	Season *p*	Size *p*	Season sig	Size sig
Coriolopsis	0.4382	0.3600	0.4637	0.3647	0.4923	0.3490	ns	ns
Hyphodontia	0.2901	0.2310	0.1354	0.3797	0.5323	0.0249	ns	*
Tulasnella	0.0367	0.0232	0.0169	0.0441	0.5238	0.2135	ns	ns
Schizopora	0.0399	0.0117	0.0379	0.0227	0.1808	0.3843	ns	ns
Malassezia	0.1082	0.0000	0.0923	0.0494	0.3140	0.6196	ns	ns
Entomophthora	0.0107	0.1888	0.1074	0.0488	0.0594	0.5671	ns	ns
Diatrype	0.0124	0.0181	0.0172	0.0122	0.5918	0.6517	ns	ns
Wallemia	0.0166	0.0514	0.0453	0.0158	0.3591	0.4542	ns	ns
Candida	0.0177	0.0144	0.0152	0.0176	0.8183	0.8829	ns	ns
Ustilago	0.0021	0.0494	0.0414	0.0009	0.2185	0.3064	ns	ns
Torula	0.0043	0.0228	0.0145	0.0081	0.1145	0.6181	ns	ns
Georgefischeria	0.0000	0.0280	0.0000	0.0186	0.0224	0.0640	*	ns
Others	0.0233	0.0011	0.0127	0.0173	0.0720	0.7715	ns	ns

Table S13. Variance inflation factor (VIF) values of environmental variables during the stepwise variable selection process prior to redundancy analysis (RDA) of bacteria communities.

Environmental variable	Step 1	Step 2	Step 3	Step 4
NH_4^+	34.86	16.54	10.26	9.89
K^+	124.32	118.46		
Cl^-	125.78	99.49	9.13	7.37
SO_4^{2-}	53.44	10.83	7.35	5.26
Temp	15.95	2.02	2.01	1.85
CO	240.69	49.09	15.99	4.70
$PM_{2.5}$	728.51			
PM_{10}	135.65	58.33	22.77	
RH	31.57	21.28	2.02	1.85
NO_3^-	52.88	21.28	10.39	5.51

Table S14. VIF values of environmental variables during the stepwise variable selection process prior to RDA of eukaryotic communities.

Environmental variable	Step 1	Step 2	Step 3	Step 4	Step 5
NH_4^+	223.90	32.97	13.87	12.65	
K^+	279.63	41.79			
Ca^+	40.71	9.69	2.53	2.48	1.35
Cl^-	326.73	45.07	9.71	7.07	6.91
NO_3^-	15.49	14.19	12.94	8.42	2.27
SO_4^{2-}	96.11	11.60	7.00	5.55	4.26
Temp	38.59	1.58	1.40	1.26	1.26
CO	146.86	14.87	14.69	4.66	4.59
$PM_{2.5}$	2361.68				
PM_{10}	800.89	24.51	22.53		
